# Bronchoscopy in Rural Areas?

**DOI:** 10.1155/2012/872327

**Published:** 2012-02-06

**Authors:** Reidar Berntsen, Erik Waage Nielsen

**Affiliations:** ^1^Department of Medicine, Helgelandssykehuset Mosjøen, 8661 Mosjøen, Norway; ^2^Department of Anesthesiology, Nordlandssykehuset Bodø, 8092 Bodø, Norway; ^3^University of Nordland, Faculty of Professional Studies, 8049 Bodø, Norway; ^4^University of Tromsø, Institute of Clinical Medicine, 9037 Tromsø, Norway

## Abstract

Quality of bronchoscopy performed by one single pulmonologist in a scarcely populated subarctic area was compared to the guidelines provided by the British Thoracic Society (BTS). 103 patients underwent bronchoscopy. Diagnostic yield was increased to 76.6% when the first bronchoscopy was supplemented by bronchial washing fluid and brush cytology and to 86.7% (BTS guidelines >80%) after a second bronchoscopy. Median time from referral to bronchoscopy was 10 days and 8 days from positive bronchoscopy to operative referral to another hospital. 1% of patients that underwent transbronchial lung biopsy had minor complications. One pulmonologist had rate of correct diagnosis based on visible endobronchial tumors that was comparable to the rates of numerous pulmonologists at larger centers performing the same procedure. Time delay was short. Rate of complications was comparable. Bronchoscopy performed by one pulmonologist alone could, in organized settings, be carried out at local hospitals in areas of scattered settlement.

## 1. Introduction

The British Thoracic Society (BTS) has given guidelines for bronchoscopy and diagnostic yield when performing bronchoscopy of visible potentially malignant lesions [1]. The guidelines are based on the practice of large bronchoscopy-performing facilities. With 16 inhabitants per square kilometer, Norway is, following Iceland, the least densely populated country in Europe. In our subarctic county the density is mere 5 pr·km^2^. We wanted to investigate whether bronchoscopic procedures performed by the only pulmonologist at a small local hospital in this county could match the demands of the British guidelines. We have mapped out the time spent from the onset of symptoms to referral, assessment, examination (bronchoscopy), operation, and further clarification at another hospital. The aim of this study was to see if the pulmonologist in question (R. Berntsen) had a diagnostic yield in percent exceeding 80, which is the limit of the BTS guidelines, and, simultaneously, if the number of complications was at an acceptable level.

## 2. Materials and Methods

The survey comprises 119 patients indicated for bronchoscopy in a nine-year period ending in 2006. Deidentified information concerning gender, age, smoking, relevant times of bronchoscopy, indication for bronchoscopy, type of local anesthetic used, and types of complications was gathered from the patient's journal and from a separate patient protocol and written out by the same nurse in pulmonology during the entire period. The pulmonologist plotted the information into an Excel spreadsheet and analyzed it by the means of autofilter functions and pivot tables.

Two nurses, of whom one specialized in pulmonology, assisted the pulmonologist during the procedure. In case of emergency an anesthetist would also be of assistance. Patients with all kinds of bleeding diatheses did not undergo bronchoscopy, and anticoagulants would have to be postponed in advance if biopsies were planned. Patients were also screened upfront by a wide panel of blood tests, ECG, spirometry, pulse oximetry. Patients with asthma would receive salbutamol inhalation before bronchoscopy. COPD patients with bronchial hyperreactivity who responded to bronchodilators would get salbutamol and ipratropium bromide before bronchoscopy. One hour after bronchoscopy with transbronchial biopsies, chest X-ray was taken to exclude pneumothorax. 

The procedure was carried out with a flexible bronchoscope. All the patients were premedicated with Hydrocodone tablets 5–10 mg and 0.6 mg subcutaneous injections of Atropine. Patients with asthma or bronchial hyperreactivity were additionally given Ventoline and Atrovent on a vaporizer. All patients were administered xylocaine locally in the throat/larynx and received an additional Xylocaine spray once the vocal cords were transited. None was initially offered intravenous midazolam but three were either administered diazepam or midazolam during the procedure. 90% of the patients were policlinical. 

## 3. Results

**Figure 1:**
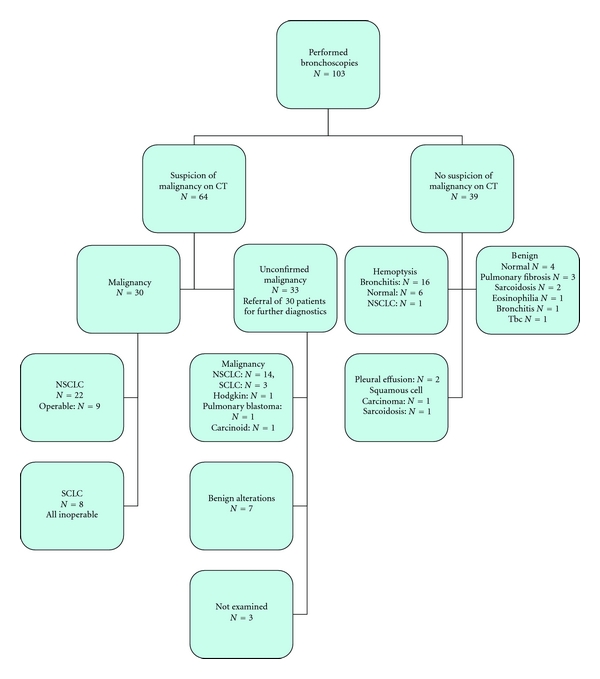


Sixteen (13%) of the 119 patients that were referred did not have a bronchoscopy, even though eight had an indication for it. Tables 1, 2, 3, 4 and 5 show the characteristics of the 103 patients that underwent bronchoscopy, their reason for referral, timeline, complications, and diagnoses. The results of the bronchoscopy and the ensuing diagnostic course are illustrated in Figure 1.

## 4. Discussion

### 4.1. Reason for Referral/Diagnosis

A diagnostic yield of 86.7% is in accord with the BTS guidelines [1]. The guidelines recommend biopsy, brush cytology, and bronchial washing fluid when a lesion is visible by bronchoscopy. In their guidelines, BTS cites a study of 5 centers that perform bronchoscopy with a diagnostic yield of malignancy by biopsy at 82% [1]. Combined use of bronchial washing fluid and brush cytology increased this yield to 87% when bronchoscopy revealed visible lesions with a malignant appearance. In our study, malignancy at first bronchoscopy was detected in 56.6% of the cases, in contrast to 44.9% in other studies [2].

In our material, the diagnostic yield of malignancy was 9% when the lesions were endoscopically invisible. This is equal to a Scottish study where the yield was at 9% [3].

Of the patients diagnosed with primary lung cancer by the pulmonologist, 31% (nine patients) were operable. Of these nine, one did not wish to have the operation. In comparison, use of surgery in patients with NSCLC in a UK region was 13% [4]. The reason for such a high number of operable patients in our material is possibly due to the short period from hospital referral to the performed bronchoscopy, but we cannot exclude this as a coincidence or due to other variables.

We had a relatively large fraction of patients undergoing bronchoscopy by indication of hemoptysis without proven pulmonic alterations by diagnostic imaging. Only one of these patients was diagnosed with malignancy. This is well in accord with textbooks in pulmonology [5].

Our hospital conducts few bronchoscopies per annum but its advantage shown in this study is that the same doctor performs nearly all these. The same doctor (R. Berntsen) carried out all but one of the bronchoscopies in our material. Larger facilities perform a greater amount of bronchoscopies but the number is divided between a larger work force of whom some at all times are under training. Also in Norway pulmonologists are generally well educated in the procedure of bronchoscopy before doing it on their own, and sufficient time to do bronchoscopy is allocated. The pulmonologist in this study also had fewer competing tasks as few patients receive mechanical ventilation in this small hospital.

### 4.2. Time Consumption

Our study proved that there was a short delay—a median of 10 days—from the hospital referral to the actual bronchoscopy, and 8 days from bronchoscopy to operative referral to another hospital. These delays are in accordance with BTS recommendations to respiratory physicians for organising the care of patients with lung cancer [6]. It is likely to believe, but hard to prove, that a prolonged period of diagnosing and treating lung cancer aggravates the prognosis [7].

### 4.3. Complications

In the BTS guidelines it is stated that flexible bronchoscopy is an extremely safe procedure if the basic precautions are taken [1]. Three retrospective studies are cited. One reports a mortality of 0.01% and serious complications in 0.08% of 24 521 procedures. Another states 0.02% mortality and 0.3% serious complications in approximately 48 000 procedures. The third study reports 0.04% mortality and 0.12% serious complications in about 40 000 procedures. A smaller prospective study of 4000 procedures, including 2000 with bronchoalveolar lavage and 173 transbronchial biopsies, revealed a higher rate of complications with 0.5% and 0.8% serious and minor complications, respectively. There were no deaths. It is further pointed out that the risk of complications (pneumothorax in 1–5% of cases and light bleeds in 9% of cases) was higher when a transbronchial biopsy was taken. The serious life-threatening complications mentioned in the BTS guidelines include respiratory depression, pneumonia, pneumothorax in need of a chest drain tube, airway obstruction, cardiorespiratory failure, arrhythmia, and pulmonary edema. Lesser, not life-threatening complications, include in declining frequency, vasovagal reactions, fever, cardiac arrhythmias, bleeds, airway obstruction, pneumothorax without need of drain tube, nausea, and vomiting.

In our study minor, non-life-threatening complications arose in 1% of cases and no serious complications of the procedure when transbronchial lung biopsy (TBB) was not performed. This is well in accord with the BTS guidelines. With TBB the rate of minor complications (bronchospasm and pneumothorax) was greater than 15%, whereas the rate for serious complications (pneumothorax in need of drain tube) was 8%. To reduce the risk of complications from flexible bronchoscopy, the BTS guidelines advise to avoid routinely atropine as premedication. The atropine is administered to reduce the bronchial secretion and suppress vagal hyperactivity. Atropine use is largely based on traditional anesthetic practice preceding rigid bronchoscopy. A study revealed that patients receiving atropine as premedication had less need of lidocaine as a local anesthetic, but there were no other apparent advantages. Premedication with anticholinergic agents may also dissolve bronchoconstriction induced by local anesthetics. This is of potential value to asthmatic patients. Atropine may cause tachycardia and can be proarrhythmogenic, cause blurry vision, trigger glaucoma, and dryness of the mouth. In our material there were no patients that presented with any of these side effects of atropine yet it is prudent to consider the BTS guidelines. Our study shows that after a thorough education by skilled pulmonologists in larger hospitals, one single pulmonologist in a small hospital can safely perform bronchoscopy by carefully selecting the patients.

### 4.4. Nonexamined Patients

The group was not clearly distinguishable from the group that underwent bronchoscopy. 16 patients were not examined. Four had an interrupted procedure because of panic attacks and laryngospasms, three had contraindications to the procedure, one was excluded based on a negative CT following an initially positive chest X-ray, in one case the diagnosis was made from a liver biopsy, one procedure was cancelled because of a faulty scope-cleaning apparatus, and one was excluded when the initially suspected malignant infiltrate proved to be benign. The seven patients with a solid indication for bronchoscopy could most likely have completed the procedure if they had been offered sedation in addition to local anesthetics, and this is now the standard. The BTS guidelines recommend that all patients should be offered sedation if there is no contraindication. Midazolam, propofol, or the combination of benzodiazepine and short-acting opiate, like alfentanil, is recommended. It is important to realize that Norway demands anesthesiologist be present when a patient receives medication causing loss of consciousness.

## 5. Conclusions

We found one pulmonologist to have rate of correct diagnosis, based on visible endobronchial tumors, comparable to the rates of numerous pulmonologists at larger centers performing the same procedure. This was also true concerning the time spent and the complications encountered. Bronchoscopy performed by one pulmonologist alone could, in organized settings, be carried out at local hospitals in areas of scattered settlement.

## Figures and Tables

**Table 1:** Description of the 103 patients who underwent bronchoscopy.

	Women	Men
Number	40	63
Median age (distribution)	69 (34–82)	66 (23–89)
Fraction smokers	29/40	58/63
Second bronchoscopy	1	5

**Table 2:** Reason of referral.

	Women	Men
Hemoptysis	10	13
Suspected malignancy	23	41
Lung consolidation without suspected malignancy	6	6
Other	1	3

**Table 3:** Time spent.

Period	No. of patients	Days median (25–75 percentile)
From symptoms to hospital referral	78	68 (30–150)
From referral to bronchoscopy	103	10 (6–25)
From bronchoscopy to surgical referral	10	8 (7–11)
From surgical referral to surgery	8	33 (27–44)
From bronchoscopy to further elaboration and treatment	30	10 (6–14)
From bronchoscopy to further elaboration and treatment in another hospital	29	38 (27–58)

**Table 4:** Complications to the 103 bronchoscopies.

	Transbronchial *N* = 13	Non-transbronchial *N* = 90
None	10	89
Minor, not life-threatening^(1)^	2 (15%)	1 (1,1%)
Serious^(2)^ (pneumothorax with drain)	1 (8%)	0

(1) BTS definition [1]: Vasovagal reaction, fever, arrhythmia, bleeds, airway obstruction, pneumothorax without the need of drain, nausea, vomiting. (2) BTS definition [1]: Respiratory depression, pneumonia, pneumothorax in need of chest drain tube, airway obstruction, cardiorespiratory arrest, arrhythmia, pulmonary edema.

**Table 5:** Diagnostic method for final diagnosis in the 53 cases of malignant pulmonary disease and other malignancy.

	*n*	%
Initial bronchoscopy	30	56,6
Second bronchoscopy	7	13,2
Transthoracic CT guided biopsy	6	11,3
Mediastinoscopy	3	5,6
Operation, open lung biopsy	3	5,6
